# Does Insufficient Sleep Increase the Risk of Developing Insulin Resistance: A Systematic Review

**DOI:** 10.7759/cureus.23501

**Published:** 2022-03-26

**Authors:** Trisha Singh, Tarig H Ahmed, Nusyba Mohamed, Mohamed S Elhaj, Zahir Mohammed, Christian N Paulsingh, Mohamed B Mohamed, Safeera Khan

**Affiliations:** 1 Internal Medicine, California Institute of Behavioral Neurosciences & Psychology, Fairfield, USA

**Keywords:** prediabetes, glucose intolerance, insulin resistance, insomnia, short sleep, sleep deprivation

## Abstract

It has been recommended that adults sleep a minimum of seven hours of sleep every night to maintain holistic health and well-being. A considerable fraction of the adult population suffers from sleep deprivation and related disorders. The stress of modern-day living may be the cause of this curtailment of sleep duration. The primary purpose of this study was to investigate the effects of reduced sleep on the development of insulin resistance and explore the possible mechanisms linking the two. We utilized databases like such as PubMed, PubMed Central (PMC), and Medical Literature Analysis and Retrieval System Online (MEDLINE) to systematically screen papers using keywords and Medical Subject Heading (MeSH) terms. A few articles were also retrieved from Cochrane Library. We applied inclusion/exclusion criteria after screening papers via title and abstracts. A quality appraisal check was doneperformed, and ten 10 related studies were strictly reviewed.

Short sleep duration was significantly associated with insulin resistance. Inflammatory markers such as C-reactive protein (CRP) and serum amyloid A (SAA), biomarkers such as glucagon-like peptide-1 (GLP-1), and circadian misalignment may play a significant role in the pathogenesis of this association. To prevent metabolic complications such as type- 2 diabetes, adequate sleep (more than seven hours per night) is required in the adult population. The causal relationship between sleep deprivation and insulin resistance is multifactorial, and further studies are warranted to understand these mechanisms better.

## Introduction and background

Sleep deprivation has reached proportions of a global pandemic in the last decade. Adults should sleep seven hours or more per night to maintain holistic health and well-being, as recommended by the American Academy of Sleep Medicine [1]. The Centers for Disease Control and Prevention (CDC) defines short sleep duration as less than seven hours of sleep per 24-hour period [2]. In the United States, 35.2% of the adult population reported being short sleepers [2]. The stress of modern-day living with ever-increasing work hours, demanding academic programs, social obligations, and the rise of electronic media might contribute to short sleep durations. This curtailment of sleep may be linked to a myriad of ill-health effects, including metabolic disturbances, hypertension, other cardiovascular diseases, decreased immunity, and increased susceptibility to infections. Some studies have even connected chronic sleep loss to cancer [3]. Research has also shown that adequate sleep is essential for optimal mental and emotional health [4].

Clinically, insulin resistance is defined as the inability of a known amount of insulin to increase glucose uptake and utilization in an individual compared to an average population [5]. In other words, the effectiveness of the hormone is reduced. As a result, the pancreas secretes greater quantities of insulin to control blood sugar levels. Among adults, the prevalence of insulin resistance ranges from 15.5% to 46.5% globally [6]. Insulin resistance is a well-established predisposing factor for the development of non-insulin-dependent diabetes mellitus [7].

Sleep deprivation and insulin resistance are both extensively prevalent worldwide, raising the question of whether insufficient sleep and insulin resistance are related. Many studies have implicated sleep loss as a risk factor for developing insulin resistance and type 2 diabetes [8,9]. However, the causal relationship between sleep deprivation and insulin resistance remains unclear. Increased levels of inflammatory markers, such as C-reactive protein (CRP), may be linked to the mechanism of how the loss of sleep may lead to prediabetes [9]. One study also suggested that serum amyloid A (SAA) may play a role in this association [10].

Further study is warranted due to the lack of coherence in the existing evidence about the pathophysiological mechanisms behind sleep deprivation as a potential risk factor for glucose intolerance and prediabetes development and progression. The rising prevalence of insulin resistance and consequent diabetes further emphasizes the importance of identifying and investigating the preventable predictors of prediabetes. Our systematic review aims to analyze further whether sleep deprivation increases the risk of developing insulin resistance and the potential mechanisms linking this association.

## Review

Methods

Protocol

We conducted this systematic review using the Preferred Reporting Items for Systematic Reviews and Meta-Analyses (PRISMA) guidelines [11].

Data Source and Strategy

We used the following databases to conduct our research: PubMed, PubMed Central (PMC), Medical Literature Analysis and Retrieval System Online (MEDLINE), and Cochrane Library. We researched the database PubMed on July 23, 2021. The search for pertinent articles was conducted using relevant concepts ("sleep deprivation" and "insulin resistance"). We then combined these concepts with keywords using the Boolean term "OR". After the application of some Medical Subject Headings (MeSH) such as "metabolism," "complications," "etiology," and "prevention and control," PubMed Search Builders were created, as shown in Table 1.

**Table 1:** Keywords and PubMed search builders

Concept	Keywords	PubMed Search Builder
Sleep deprivation	Sleep loss, sleep debt, insomnia, lack of sleep	Sleep deprivation OR insomnia OR lack of sleep OR sleep loss OR sleep debt OR ("Sleep Deprivation"[Mesh]) AND ( "Sleep Deprivation/complications"[Majr] OR "Sleep Deprivation/metabolism"[Majr] )
Insulin resistance	Glucose intolerance	Insulin resistance OR glucose intolerance OR ( "Insulin Resistance/etiology"[Majr] OR "Insulin Resistance/prevention and control"[Majr] )

The keywords were pooled using the Boolean term "OR" and were combined with their corresponding search builder acquired from PubMed using MeSH terms.

Furthermore, restrictions to MeSH-major topics were applied. All concepts and keywords were combined into a final search strategy using the Boolean term "AND," as shown in Table 2.

**Table 2:** Full MeSH strategy

Full MeSH Strategy	Number of Articles
Sleep deprivation OR insomnia OR lack of sleep OR sleep loss OR sleep debt OR ("Sleep Deprivation"[Mesh]) AND ( "Sleep Deprivation/complications"[Majr] OR "Sleep Deprivation/metabolism"[Majr] ) AND Insulin resistance OR glucose intolerance OR ( "Insulin Resistance/etiology"[Majr] OR "Insulin Resistance/prevention and control"[Majr] )	Before filters applied = 29,949; After filters applied = 1,244 (filters: articles published in the last five years, Articles published in the English language, free full text, adults 19-44 years and middle-aged 45-64 years)

The PubMed search builders were combined into one comprehensive search strategy using the boolean term "AND".

Screening of Articles

After obtaining the relevant articles from all the databases, we removed the duplicates. Then, the articles were screened based on title, abstract, and reading full-text articles. Finally, 10 articles were short-listed and subjected to a quality appraisal.

Inclusion and Exclusion Criteria

The literature search was conducted to identify relevant studies that examine the role of sleep deprivation in the development of insulin resistance. Inclusion criteria were studies conducted in the adult population and between ages 18 and 65, and published in English as full-text papers in the past five years. Studies conducted in the pediatric and geriatric population, unpublished literature, and grey literature were excluded.

Quality Appraisal Tools

We conducted the quality assessment of the short-listed articles using the tools displayed in Table 3. Only articles satisfying >70% of the quality parameters were included in the systematic review.

**Table 3:** Quality appraisal tools SANRA, Scale for Assessment of Narrative Review Articles; SYRCLE, Systematic Review Center for Laboratory animal Experimentation

Type of Study	Quality Appraisal Tool
Randomized controlled trials	Cochrane Bias Assessment Tool
Non-randomized clinical trials and observational studies	New Castle Ottawa Tool
Research papers without a clear methods section	SANRA Checklist
Animal studies	SYRCLE

Results

**Figure 1:**
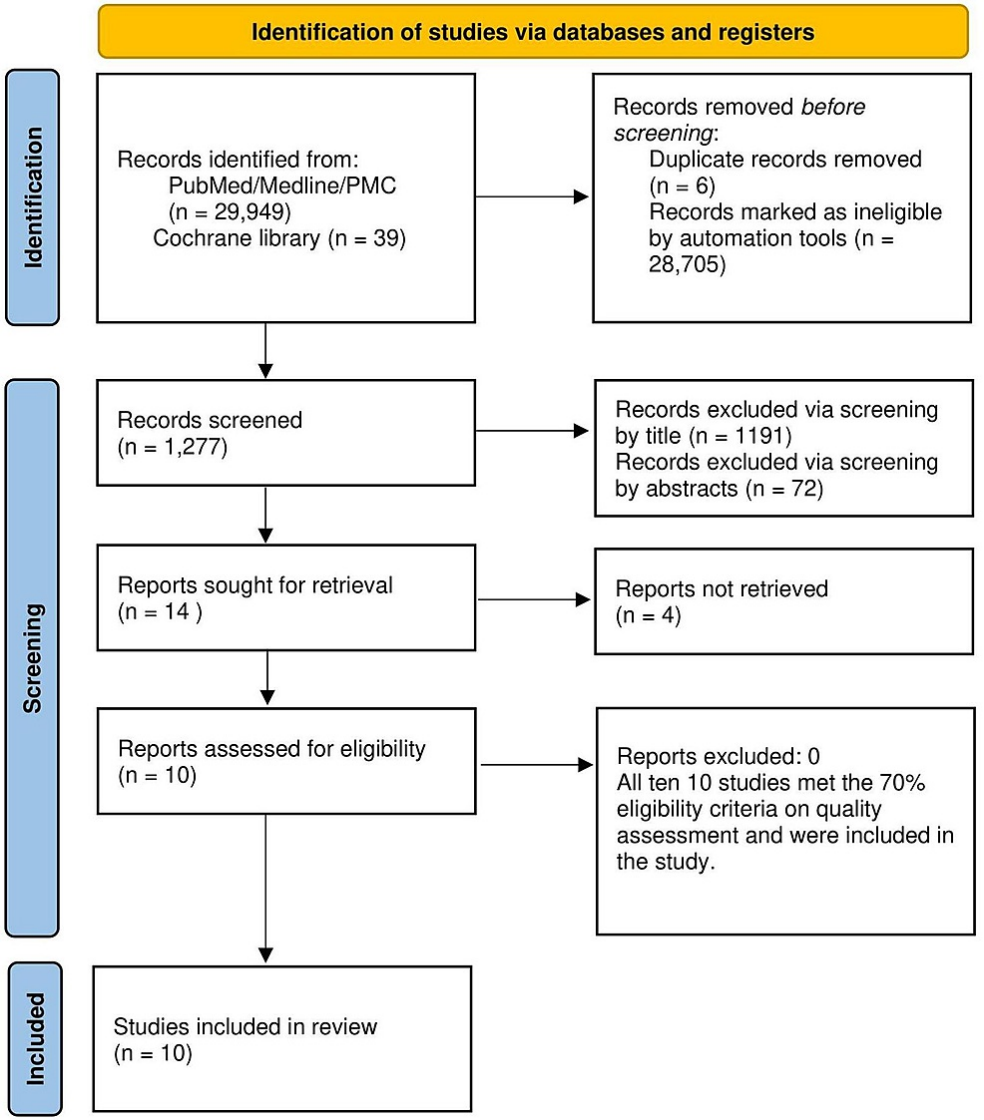
PRISMA flow diagram depicting the article selection process PRISMA, Preferred Reporting Items for Systematic Reviews and Meta-Analyses

We used four databases to search for relevant articles: PubMed, PMC, MEDLINE, and Cochrane Library. The research yielded 29,988 articles. After we removed duplicates, 29,982 articles remained. Inclusion/exclusion criteria were applied, resulting in 1,277 articles. Papers were then screened using title relevance, abstracts, and full texts, which generated 10 studies that satisfactorily met the quality assessment. Figure 1 illustrates the search strategy in the form of a PRISMA flow diagram.

We performed a quality assessment for the 10 studies using various tools: Cochrane Risk of Bias Tool (n=4), New Castle Ottawa Tool (n=4), Scale for Assessment of Narrative Review Articles (SANRA) Checklist (n=1), and Systematic Review Center for Laboratory animal Experimentation (SYRCLE) (n=1). All 10 articles were included in this systematic review as all satisfied the cutoff (>70%).

Table 4 summarizes the data extracted from the articles included in this systematic review.

**Table 4:** Summary of data extracted from articles CRP, C-reactive protein; SAA, serum amyloid A; PSD, partial sleep deprivation; CPIR, cephalic phase insulin release; RCT, randomized controlled trial; AI/ANs, American Indians/Alaska Natives; OSA, obstructive sleep apnea; GLP-1, glucagon-like peptide 1; NEFA, non-esterified fatty acid

Author and Year of Publication	Purpose of Study	Number of Patients	Type of Study	Result	Conclusion
Iyegha et al., 2019 [9]	They explored the association between glucose metabolism and sleep quality among people with prediabetes and the possible mechanisms linking poor sleep to glucose intolerance.	155	Observational	Compared to those in the standard glucose group, more people with prediabetes suffered from poor sleep quality. The CRP levels were elevated in the prediabetes group compared to the standard glucose group. A positive correlation was also found between disturbed sleep and CRP.	Poor sleep quality is associated with prediabetes. Increased levels of CRP may be a likely cause underlying the association between poor sleep and prediabetes.
de Oliveira et al., 2017 [10]	They investigated whether SAA production during sleep restrictions contributes to the development of associated comorbidities related to sleep loss.	Six mice, 30 humans	Animal	SAA and endotoxin production was increased to approximately four times normal in case of sleep restriction.	Sleep deprivation precipitates SAA production in healthy mice and humans. Elevated levels of SAA may be a part of the signaling cascade connecting sleep loss to its associated ill-health effects such as obesity and type 2 diabetes.
Cedernaes et al., 2016 [12]	This study investigated the effect of a single night of PSD on fasting insulin sensitivity and CPIR in humans.	16	RCT	PSD was significantly associated with higher peripheral insulin resistance than a night of complete sleep.	This study evidenced that sleeping at least seven hours per night may decrease the risk of developing insulin resistance. They also suggest that an altered CPIR (and subsequent disrupted autonomic signaling, which regulates the CPIR) is probably not likely to be the mechanism through which one night of PSD causes metabolic abnormalities.
So-ngern et al., 2019 [13]	This study explored if extending sleep duration for two weeks would improve glucose tolerance in habitually sleep-deprived individuals.	21	RCT	No significant effects of extending sleep duration were found on any metabolic outcomes.	Glucose tolerance improved only in those individuals who could objectively increase their sleep duration to more than six hours per night. Their findings suggested that a necessary amount of sleep is required to improve metabolism.
Nuyujukian et al., 2016 [14]	This study examined the relationship between self-reported sleep duration and the incidence of type 2 diabetes in a population sample of AI/ANs with prediabetes.	1,899	Observational	The incidence of diabetes was higher among short sleepers (≤ six hours per night) than those sleeping seven hours.	Short sleep duration was significantly associated with increased diabetes risk and reduced weight loss.
Bowman et al., 2019 [15]	This study examined the prospective association between polysomnography and self-reported sleep deprivation with metabolic syndrome.	145	Observational	None of the objectively or subjectively assessed sleep indices were associated with metabolic syndrome.	Cross-sectional associations among objectively and subjectively assessed sleep with metabolic syndrome were insignificant.
Wang et al., 2017 [16]	This study aimed to identify if metabolic syndrome was associated with insomnia.	8,017	Observational	Insomnia was not significantly associated with dysglycemia.	The association between insomnia and dysglycemia was not significant. However, those who had insomnia were more susceptible to increased severity of metabolic abnormalities.
Reutrakul et al., 2017 [17]	This study scrutinized the relationship between sleep quality, duration, OSA, and GLP-1 regulation in participants with abnormal glucose tolerance.	71	Observational	GLP-1 did not vary among those sleeping ≤ 5.75 hours, > 5.75 to < 6.5 hours, or ≥ 6.5 hours per night.	OSA severity, but not habitual sleep duration or quality, was associated with lower GLP-1 response to glucose challenge in patients with abnormal glucose tolerance.
Ness et al., 2019 [18]	This study investigated whether sleep restriction affected NEFA suppression and whether recovery sleep for two nights is enough to rehabilitate metabolic health.	15	RCT	Sleep restriction significantly suppressed NEFA rebound levels. NEFA levels returned to baseline after recovery sleep.	NEFA metabolism was significantly affected by sleep restriction. This study demonstrated that two nights of recovery sleep is insufficient to improve glucose metabolism.
Qian et al., 2018 [19]	This study explored the different effects of the circadian system and circadian misalignment on insulin sensitivity and beta-cell function.	14	RCT	The results demonstrated that the inherent biological circadian system and circadian misalignment affect insulin sensitivity via independent processes.	The biological circadian system reduced glucose sensitivity in the biological evening in comparison to the biological morning by affecting beta-cell function. Circadian misalignment did not affect beta-cell function but reduced glucose tolerance by reducing insulin sensitivity.

Discussion

*Is Sleep Deprivation Associated With Insulin Resistance*?

Many articles included in this systematic review concluded a significant association between short sleep duration and insulin resistance. Cedernaes et al. found that one night of partial sleep deprivation compared to an entire night's sleep resulted in appreciably increased peripheral insulin resistance [12]. A crossover study by So-ngern et al. demonstrated that glucose tolerance improved by extending sleep duration in regularly sleep-deprived individuals and in those who could quantitatively increase their sleep duration to more than six hours per night [13]. Nuyujukian et al. conducted a study in a national sample of American Indians and Alaskan natives with prediabetes, inspecting the association between self-reported sleep duration and the incidence of diabetes, and found that short sleep duration (defined as < six hours per night) significantly elevated the risk of developing diabetes [14].

Some studies evaluated the association between sleep and components of metabolic syndrome. Bowman et al. conducted a study in which they measured sleep - both objectively and subjectively - and correlated it with the risk of developing metabolic syndrome. In particular, they found that longer sleep latency was significantly associated with higher fasting glucose levels in participants who self-reported sleep duration. However, polysomnography measured sleep duration was not significantly associated with any of the components of metabolic syndrome [15]. In most other studies, sleep duration was self-reported by participants. This is a more subjective assessment of sleep duration rather than utilizing a more objective sleep measurement, such as polysomnography. This may be subject to reporting and measurement bias. Studies have also shown that self-reported sleep duration tends to be longer than objectively measured sleep duration [13]. Thus, there is scope for future studies and trials to investigate further the relationship between sleep duration and its metabolic impacts by utilizing objective methods for measuring sleep duration (e.g., polysomnography).

Wang et al. reported that insomnia was independently associated with raised blood pressure and low high-density lipoprotein cholesterol (HDL-c) levels but was not associated with other components of metabolic syndrome such as dysglycemia, high triglyceride levels, and central adiposity. However, they did find that insomniacs were more prone to increased severity of metabolic abnormalities [16]. Only the research by Wang et al. reported gender differences in the association between insomnia and metabolic syndrome. They observed a significant association between insomnia and metabolic syndrome in males and the middle-aged population [16].

The study conducted by Cedernaes et al. included only male participants and observed a significant relationship between sleep deprivation and peripheral insulin resistance [12]. On the other hand, So-ngern et al. had a majority of female participants as compared to males [13]. No studies exclusively investigated the relationship between sleep loss and insulin resistance in the female population. Further studies may be warranted to explore this relationship and identify gender differences. This may also impact the pathophysiology of how sleep deprivation may contribute to the development of insulin resistance, likely due to differences in estrogen levels in the male and female populations.

Not many studies investigated the relationship between long sleep duration and metabolic derangements, except Nuyujukian et al.’s study, which found that long sleep duration was not associated with increased diabetes risk [14]. More studies are warranted to investigate the health impacts of long sleep duration.

In most studies, the follow-up time between measuring habitual short sleep and incidence of prediabetes was relatively short. This may impact the true nature of this relationship due to the lack of sufficient time gap for the incidence of prediabetes. Also, most studies were limited to racial and ethnic groups, thus limiting the generalizability of the results. Further studies should aim to mitigate this selection bias and select a more globally representative population.

Pathophysiology Linking Sleep Deprivation and Insulin Resistance

Even though several studies have suggested an association between sleep deprivation and insulin resistance, there is no clear understanding of its mechanism. Some studies suggest that the mechanism may be related to increased inflammatory markers in sleep-deprived individuals. For example, a cross-sectional study showed higher CRP levels in a group of patients with prediabetes compared to those with standard glucose tolerance [9]. Additionally, they found a positive correlation between sleep disturbance and CRP levels. These findings concluded that elevated CRP levels might be a probable underlying mechanism of the association between prediabetes and short sleep duration [9]. In another study, de Oliveira et al. investigated whether the production of SAA during sleep restriction contributes to the pathogenesis of the comorbid conditions that occur due to sleep loss. They found elevated SAA levels in mice subjected to sleep restriction for 15 days or sleep deprivation for 72 hours. Metabolic endotoxemia was also a significant finding in those subjected to sleep restriction. They also found increased plasma levels of SAA in healthy human participants who were subjected to two nights of total sleep deprivation. After one day of sleep recovery, the SAA levels returned to baseline [10]. These findings inferred that increased levels of SAA are probably a part of the pathophysiology that links sleep loss to its associated comorbidities such as obesity and type 2 diabetes [10].

On the other hand, Reutrakul et al. studied the relationship between sleep and glucagon-like peptide-1 (GLP-1) regulation in people with abnormal glucose tolerance. They found that increasing obstructive sleep apnea severity was associated with a lower GLP-1 response to glucose tolerance [17]. These findings demonstrate that this could also be a possible mechanism by which obstructive sleep apnea, and hence sleep loss, affects glucose metabolism.

Insulin resistance in adipocytes disrupts non-esterified fatty acid (NEFA) metabolism. A randomized controlled trial investigated whether sleep restriction affected NEFA suppression after administering an intravenous glucose tolerance test and whether weekend recovery sleep (two nights) was enough to rehabilitate metabolic health. They found that sleep restriction impacted NEFA metabolism and demonstrated that two nights of recovery sleep might not be adequate to restore optimum glucose control [18].

Thus, one can see that there are several inflammatory and biomarkers that have been implicated in the pathogenesis of reduced insulin sensitivity due to loss of sleep. However, due to a lack of coherence of findings among studies, further study is warranted to investigate this pathway and understand the exact mechanism through which this association is linked. Understanding this process can help us clinically manage patients better and establish the causal relationship between sleep loss and impaired glucose tolerance.

Another piece of the puzzle could be circadian misalignment and inadequate light exposure. A study conducted by Qian et al. concluded that the circadian system reduces glucose tolerance in the biological morning by decreasing dynamic and static beta-cell function. In comparison, circadian misalignment reduced glucose tolerance mainly by reducing insulin sensitivity, not affecting beta-cell function [19]. This could also be a significant contributing factor. Another typical response to physiological stressors is the release of cortisol into the bloodstream. A study conducted by Wright et al. concluded that acute total sleep deprivation leads to increased cortisol levels [20]. Studies in the future can help evaluate sleep deprivation and the rise in cortisol levels as a potential mechanistic link with the development of glucose intolerance. Future studies can also tie in the relationship between circadian misalignment, cortisol levels, and the inflammatory markers deranged due to sleep loss, and how all these factors contribute to the development of insulin resistance.

There were several limitations of this study that should be addressed. Most studies in this systematic review measured sleep duration and quality by self-reported questionnaires that may be subject to reporting and recall bias. Future studies could incorporate objective sleep measurements through polysomnograms and self-reported sleep to help mitigate this. Secondly, many studies had participants limited to specific geographical areas, limiting the generalizability of the results.

## Conclusions

This systematic review suggests a significant association between sleep deprivation and insulin resistance. Several possible mechanisms that may link this association were also studied. There seems to be a significant implication of inflammatory markers such as CRP and SAA in the causal relationship between sleep loss and glucose intolerance. Other metabolic markers such as GLP-1 and NEFA metabolism may also be implicated. Our findings suggest that adequate sleep is necessary for maintaining proper metabolic health to prevent long-term complications such as type 2 diabetes. Future studies may investigate this association further to help establish a causal relationship. This will help us emphasize the importance of adequate sleep to our patients and help manage them better. Sleep deprivation is a preventable risk factor that can help reduce the incidence of metabolic complications.

## References

[1] Watson NF, Badr MS, Belenky G (2015). Recommended amount of sleep for a healthy adult: a joint consensus statement of the American Academy of Sleep Medicine and Sleep Research Society. J Clin Sleep Med.

[2] (2021). Data and Statistics. Short Sleep Duration Among US Adults. https://www.cdc.gov/sleep/data_statistics.html.

[3] (2021). Brain Basics: Understanding Sleep. https://www.ninds.nih.gov/Disorders/patient-caregiver-education/understanding-sleep.

[4] Walker MP, van der Helm E (2009). Overnight therapy? The role of sleep in emotional brain processing. Psychol Bull.

[5] Lebovitz HE (2001). Insulin resistance: definition and consequences. Exp Clin Endocrinol Diabetes.

[6] Fahed M, Abou Jaoudeh MG, Merhi S, Mosleh JM, Ghadieh R, Al Hayek S, El Hayek Fares JE (2020). Evaluation of risk factors for insulin resistance: a cross sectional study among employees at a private university in Lebanon. BMC Endocr Disord.

[7] Lillioja S, Mott DM, Spraul M (1993). Insulin resistance and insulin secretory dysfunction as precursors of non-insulin-dependent diabetes mellitus. Prospective studies of Pima Indians. N Engl J Med.

[8] Spiegel K, Knutson K, Leproult R, Tasali E, Van Cauter E (2005). Sleep loss: a novel risk factor for insulin resistance and type 2 diabetes. J Appl Physiol (1985).

[9] Iyegha ID, Chieh AY, Bryant BM, Li L (2019). Associations between poor sleep and glucose intolerance in prediabetes. Psychoneuroendocrinology.

[10] de Oliveira EM, Visniauskas B, Tufik S, Andersen ML, Chagas JR, Campa A (2017). Serum amyloid a production is triggered by sleep deprivation in mice and humans: is that the link between sleep loss and associated comorbidities?. Nutrients.

[11] Page MJ, McKenzie JE, Bossuyt PM (2021). The PRISMA 2020 statement: an updated guideline for reporting systematic reviews. Syst Rev.

[12] Cedernaes J, Lampola L, Axelsson EK (2016). A single night of partial sleep loss impairs fasting insulin sensitivity but does not affect cephalic phase insulin release in young men. J Sleep Res.

[13] So-Ngern A, Chirakalwasan N, Saetung S, Chanprasertyothin S, Thakkinstian A, Reutrakul S (2019). Effects of two-week sleep extension on glucose metabolism in chronically sleep-deprived individuals. J Clin Sleep Med.

[14] Nuyujukian DS, Beals J, Huang H, Johnson A, Bullock A, Manson SM, Jiang L (2016). Sleep duration and diabetes risk in American Indian and Alaska Native participants of a lifestyle intervention project. Sleep.

[15] Bowman MA, Duggan KA, Brindle RC, Kline CE, Krafty RT, Thayer JF, Hall MH (2019). Prospective associations among objectively and subjectively assessed sleep and the metabolic syndrome. Sleep Med.

[16] Wang Y, Jiang T, Wang X (2017). Association between insomnia and metabolic syndrome in a Chinese Han population: a cross-sectional study. Sci Rep.

[17] Reutrakul S, Sumritsopak R, Saetung S, Chanprasertyothin S, Anothaisintawee T (2017). The relationship between sleep and glucagon-like peptide 1 in patients with abnormal glucose tolerance. J Sleep Res.

[18] Ness KM, Strayer SM, Nahmod NG, Chang AM, Buxton OM, Shearer GC (2019). Two nights of recovery sleep restores the dynamic lipemic response, but not the reduction of insulin sensitivity, induced by five nights of sleep restriction. Am J Physiol Regul Integr Comp Physiol.

[19] Qian J, Dalla Man C, Morris CJ, Cobelli C, Scheer FA (2018). Differential effects of the circadian system and circadian misalignment on insulin sensitivity and insulin secretion in humans. Diabetes Obes Metab.

[20] Wright KP Jr, Drake AL, Frey DJ, Fleshner M, Desouza CA, Gronfier C, Czeisler CA (2015). Influence of sleep deprivation and circadian misalignment on cortisol, inflammatory markers, and cytokine balance. Brain Behav Immun.

